# Genetic diversity analysis in a set of Caricaceae accessions using resistance gene analogues

**DOI:** 10.1186/s12863-014-0137-0

**Published:** 2014-12-10

**Authors:** Samik Sengupta, Basabdatta Das, Pinaki Acharyya, Manoj Prasad, Tapas Kumar Ghose

**Affiliations:** Division of Plant Biology, Bose Institute, Main Campus, 93/1 A.P.C. Road, Kolkata, 700009 West Bengal India; Department of Horticulture, Institute of Agricultural Science, University of Calcutta, 35, Balligunge Circular Road, Kolkata, 700029 West Bengal India; National Institute of Plant Genome Research (NIPGR), Aruna Asaf Ali Marg, New Delhi, 110067 India

**Keywords:** *Carica papaya*, *Vasconcellea* sp, DNA homologues, Rice BLB genes

## Abstract

**Background:**

In order to assess genetic diversity of a set of 41 Caricaceae accessions, this study used 34 primer pairs designed from the conserved domains of bacterial leaf blight resistance genes from rice, in a PCR based approach, to identify and analyse resistance gene analogues from various accessions of *Carica papaya*, *Vasconcellea goudotiana*, *V. microcarpa*, *V. parviflora*, *V. pubescens*, *V. stipulata* and, *V. quercifolia* and *Jacaratia spinosa*.

**Results:**

Of the 34 primer pairs fourteen gave amplification products. A total of 115 alleles were identified from 41 accesions along with 12 rare and 11 null alleles. The number of alleles per primer pair ranged from 4 to 10 with an average of 8.21 alleles/ primer pair. The average polymorphism information content value was 0.75/primer. The primers for the gene Xa1 did not give any amplification product. As a group, the Indian Carica papaya accessions produced a total of 102 alleles from 27 accessions. The similarity among the 41 accessions ranged from 1% to 53%. The dendrogram made from Jaccard’s genetic similarity coefficient generated two major clusters showing that the alleles of *Jacaratia spinosa* and *Vasconcellea* accessions were distinctly different from those of *Carica papaya* accessions. All the alleles were sequenced and eleven of them were allotted accession numbers by NCBI. Homology searches identified similarity to rice BLB resistance genes and pseudogenes. Conserved domain searches identified gamma subunit of transcription initiation factor IIA (TFIIA), cytochrome P450, signaling domain of methyl-accepting chemotaxis protein (MCP), Nickel hydrogenase and leucine rich repeats (LRR) within the sequenced RGAs.

**Conclusions:**

The RGA profiles produced by the 14 primer pairs generated high genetic diversity. The RGA profiles identified each of the 41 accessions clearly unequivocally. Most of the DNA sequences of the amplified RGAs from this set of 41 accessions showed significant homology to the conserved regions of rice bacterial leaf blight resistance genes. These information can be used in future for large scale investigation of tentative disease resistance genes of *Carica papaya* and other Caricaceae genus specially *Vasconcellea.* Inoculation studies will be necessary to link the identified sequences to disease resistance or susceptibility.

**Electronic supplementary material:**

The online version of this article (doi:10.1186/s12863-014-0137-0) contains supplementary material, which is available to authorized users.

## Background

Papaya (*Carica papaya* L.), is one of the major fruit crops cultivated in tropical and sub-tropical zones. Over 6.8 million tonnes of this fruit are produced worldwide with India in the lead having an annual output of about 3 million tonnes [1]. Other leading producers are Brazil, Mexico, Nigeria, Indonesia, China, Peru, Thailand and Philippines. Papaya is eaten both fresh and cooked, and is processed into pickles, jams, candies, fruit drinks and juices. Papain, an enzyme purified from papaya latex, is extracted for export. The enzyme is used in medicine, breweries, textile and leather processing industries. Susceptibility to insect, pest and diseases are the major constraints limiting papaya production. Papaya ringspot virus (PRSV), *Xanthomonas* fruit rot, black spot, die back and root rot cause huge crop loss each year. The structural makeup and functional mechanisms of genes that confer disease resistance in *Carica papaya* is largely unknown and only a few genetic markers linked to resistance genes have been identified [2-5]. Although bio-engineering efforts have been successful in controlling PRSV [6] and improved agricultural practices like application of pesticides and nutritional supplements have been used in disease control of papaya; no durable solution is available due to the breakdown of resistance by high pathogenic variability. *Vasconcellea,* a related genus from the family Caricaceae, has the potential as a source of novel genes for quality traits and disease resistance especially against papaya ringspot virus [7,8]. Resistances to several other diseases which affect *Carica papaya* have also been identified in the *Vasconcellea* genepool, including: resistance to black spot, (*V. cundinamarcensis*) [7]; die back, (*V. parviflora*) [7]; and root rot, (*V. goudotiana*) [7]. However hybridization between *Carica papaya* and *Vasconcellea* have been largely limited by post-zygotic instabilities, including embryo abortion and infertility of the hybrids [7,8]; thus presenting a significant barrier for the successful introgression of desirable disease resistance traits into C. *papaya*.

The susceptibility of papaya to diseases coupled with the difficulty in producing viable intergeneric crosses has lead to the adoption of molecular biology tools, PCR-based strategies and in-silico genomic evaluation of defense gene homologs, as a means for crop improvement and search of naturally occurring resistance in existing genotypes of papaya and related species [9]. With the publication of the 372 Mb draft sequence of the papaya genome [10,11], defense associated nucleotide-binding site (NBS)-encoding genes have been identified. Majority of the plant disease resistance proteins identified to date belong to a limited number of classes, of which those containing nucleotide-binding site (NBS) motifs are the most common. Amaral et al. [12] used the primer combination P1b and RNBS-D [13] to amplify RGAs in *Carica papaya* transgenic variety Embrapa PTP18 and *Vasconcellea cauliflora.* Forty eight clones were sequenced from each of the two species and the only RGA that was identified was from *Carica papaya* transgenic variety Embrapa PTP18. This RGA showed homology to the putative disease resistant protein RGA3 of *Solanum bulbocastanum* (gb|AAP45165.1|). Detailed in-silico analysis of the putative resistance genes (R-genes) identified by Ming et al. [11] have been done by Porter et al. [9]. They found that despite having a significantly larger genome than *Arabidopsis thaliana*, papaya has fewer NBS genes, belonging to both Toll/interleukin-1 receptor (TIR) and non-TIR subclasses. They also proposed that Papaya NBS gene family shares most similarity with *Vitis vinifera* homologs, but seven non-TIR members with distinct motif sequence represents a novel subgroup.

Although the order of plant disease resistance genes is not syntenic across taxa, majority of the defence related genes are structurally and functionally conserved across most plant species and the proteins coded have been grouped into various classes [14-16]. Synteny is the maintenance of the ordered sequence or the relative positions of the genes on the chromosome across species. With the increased availability of plant genome sequence information, syntenic relationships among the various taxa are being gradually elucidated. Studies have revealed that the gene families encoding transcription factors are syntenic throughout the angiosperm kingdom while others are subject to various aberrations [17]. Abrouk et al [18] analysed monocot synteny using rice as the reference genome and found that on the basis of short conserved sequence regions 77% of the genes were conserved among the five cereal genomes of rice, maize, wheat, *Sorghum* and *Brachypodium*. Similar analysis of eudicot synteny with grape as the reference genome showed 77% gene conservation between Arabidopsis, grape, poplar, soybean and papaya. Synteny has also been found between rice and *Arabidopsis* [19]. There are no reports of synteny between rice and papaya as of yet. However this experimentation has been based on the probable structural and functional conservation of disease resistance genes between rice and papaya.

Using degenerate PCR primers designed from the various classes of disease resistance, a number of workers like Leister et al. [20], Kanazin et al. [21] and Yu et al. [22] have developed a targeted technique for isolating homologous genes and DNA sequences. The term RGA (resistance gene analog) is used to denote such cloned homologous gene sequences for which no function has yet been assigned in the plant species [23]. Once found, the RGA can be used as probe to screen BAC or cDNA libraries, as a marker to be applied in marker assisted selection and to obtain resistance by their over expression in the plant genome.

Rice is the model monocot. Its genome has been sequenced and information regarding the structure and function of its disease resistance genes, including those against bacterial leaf blight (BLB) are publicly available [24-32]. BLB is caused by the vascular pathogen *Xanthomonas oryzae* pv. *oryzae* (*Xoo*), a gammaproteobacteria. It is one of the most serious diseases leading to crop failure in rice growing countries. Xoo enters rice leaves typically through the hydathodes at the leaf margin, multiplies in the intercellular spaces of the underlying epithelial tissue, and moves to the xylem vessels to cause systemic infection [25]. Rice Bacterial leaf blight (BLB) resistance genes *Xa1* and *Xa21* belongs to the CC/NBD/LRR (coiled coil/nucleotide binding domain/leucine rich repeat) [31] and extracellular LRR/kinase domain classes [27] respectively. The BLB resistance gene *xa5* is a transcription factor and *Xa26* codes for a receptor kinase like protein. A signal-anchor-like sequence is predicted at the amino (N)-terminal region of BLB resistance gene *Xa27* and it localizes to the apoplast. The previous attempt to isolate and identify RGAs used degenerate primers designed by Bertioli [13] using a protein alignment of L6 rust R-gene (resistance gene) from *Linum usitatissimum*, R-gene *N* against tobacco mosaic virus from *Nicotiana glutinosa*, gene *NL25* from *Solanum tuberosum* mRNA, gene *RPS5* of *A. thaliana* for resistance to *Pseudomonas syringae*, R-gene *Mi-1* against nematodes and aphids from *Lycopersicon esculentum*, and gene *Rpp8* of *A. thaliana*; and by Kanazin [21] using the conserved P-loop sequence. However attempts to identify RGAs using primers developed from known resistance genes from rice was not done before and that is what we have tried to do in this study.

Most Genetic diversity studies use DNA primers that are from random genomic locations. While, genetic diversity studies using targeted genic sequences could be more informative, useful and valuable. Das et al. [33] had designed 34 pairs of primers from the conserved motifs of 6 bacterial leaf blight resistance genes of Oryza sativa – *Xa1, xa5, Xa21, Xa21(A1), Xa26* and *Xa27*, for the assessment of genetic diversity amongst rice acccessions. In this study we have used those 34 primer pairs to identify RGAs in 41 accessions of *Carica papaya*, *Vasconcellea* sp and *Jacaratia spinosa*. The other objectives of this study were, to obtain the genetic relationship amongst the 41 Caricaceae accessions using the polymorphism of the amplified DNA bands using statistical methods, and to analyze the sequences of the obtained DNA bands for the presence of homology and conserved domains.

## Method

### Plant materials

The germplasm set in this study included 1 accession each from 27 Indian and 7 foreign commercially popular *Carica papaya* cultivars, 1 accession each of *V. goudotiana*, *V. microcarpa*, *V. parviflora*, *V. pubescens*, *V. stipulata* and *V. quercifolia* and 1 accession of South American tree species *Jacaratia spinosa.* The collection was maintained at the experimental farm of Acharya J.C. Bose Biotechnology Innovation Centre, Bose Institute at Madhyamgram, West Bengal, India. Fully expanded fourth leaf from the top was used as source material for genomic DNA isolation. The category, cultivar name, source and number of accessions used in this study for each accession are given in Table 1.

**Table 1:** **Name, category, source and number of accessions of each cultivar used in this study**

**Indian** ***Carica papaya*** **cultivars**			
**Cultivar name**	**Category**	**Source**	**Number of accessions**
Ambasa local (RT2)	Local adaptive genotype	ICAR, Tripura	1
Bangalore Dwarf	Local adaptive genotype	Pvt. seed company	1
CO 1	Principal genotype	ICAR, Tripura	1
CO 2	Principal genotype	Pvt. seed company	1
CO 3	Principal genotype	Pvt. Seed company	1
CO 4	Principal genotype	OUAT	1
CO 5	Principal genotype	IIHR	1
CO 6	Principal genotype	IIHR	1
CO 7	Principal genotype	Pvt. Seed company	1
Coorg Honey Dew	Local adaptive genotype	IIHR	1
Farm Selection -1	Local adaptive genotype	ICAR, Tripura	1
Honey Dew	Minor genotype	IIHR	1
Madhu	Local adaptive genotype	ICAR, Tripura	1
Orissa local	Local adaptive genotype	Pvt. seed company	1
Pant 2	Local adaptive genotype	ICAR, Tripura	1
PAU Selection	Local adaptive genotype	TNAU	1
Pusa Dwarf	Principal genotype	TNAU	1
Pusa Giant	Principal genotype	TNAU	1
Pusa Nanha	Principal genotype	TNAU	1
Ranchi	Minor genotype	Pvt. seed company	1
Ranch Dwarf	Local adaptive genotype	TNAU	1
Red Indian	Principal genotype	IIHR	1
RT1	Local adaptive genotype	IIHR	1
Shillong	Local adaptive genotype	IIHR	1
Surya	Principal genotype	Pvt. seed company	1
Washington	Local adaptive genotype	IIHR	1
Yellow Indian	Principal genotype	Pvt. seed company	1
**Foreign** ***Carica papaya*** **cultivars**			
**Cultivar name**	**Category**	**Source**	**Number of accessions**
Hortus Gold	South African cultivar	Pvt. seed company	1
Kapoho	Hawaiian cultivar	USDA	1
Solo papaya 109	Hawaiian cultivar	USDA	1
Sunrise Solo	Hawaiian cultivar	USDA	1
Taiwan	F1 hybrid Tainung series	Pvt. seed company	1
Taiwan Red Lady	F1 hybrid Tainung series	Pvt. seed company	1
Waimanlo	American cultivar (Florida)	Pvt. seed company	1
**Other** ***Caricaceae*** **species**			
**Cultivar name**	**Category**	**Source**	**Number of accessions**
*Jacartia spinosa*	Related genus	USDA	1
*Vasconcellea gouditiana*	Highland papaya	USDA	1
*Vasconcellea microcarpa*	Highland papaya	USDA	1
*Vasconcellea parviflora*	Highland papaya	USDA	1
*Vasconcellea pubescens*	Highland papaya	USDA	1
*Vasconcellea stipulata*	Highland papaya	USDA	1
*Vasconcellea quercifolia*	Highland papaya	USDA	1

ICAR – Indian Council of Agricultural Research, IIHR – Indian Institute of Horticultural Research, OUAT- Orissa University of Agriculture and Technology, TNAU – Tamil Nadu Agriculture University, USDA – United States Department of Agriculture.

### Designing primers for bacterial leaf blight resistance

Thirty four primer pairs were designed from publicly available sequences of six rice bacterial leaf blight resistance genes using the software BatchPrimer3 (http://probes.pw.usda.gov/batchprimer3). The forward and reverse primers for the markers were coded BDTG1 to BDTG34. The primers were designed to include only the exons and so as to amplify about 500 to 700 base pairs [33]. Details of the markers are given in Table 2.

**Table 2:** **Details of the primers used**

**Marker name**	**Gene**	**Protein**	**Ann temp**	**Exon no.**	**Expected size of amplification product in rice in bp**	**Forward primer**	**Reverse primer**
BDTG 1	*Xa1*	P LOOP	59.8	1	508	5′ -ATTAATCCACGACGACCAGG – 3′	5′ -GTAGCACAAGCACCTCCTCC – 3′
BDTG 2	”	KINASE 2	60	2	429	5′ -GAGGAGGTGCTTGTGCTACAG – 3′	5′ -GGCACTGGCATTACCTTGAT – 3′
BDTG 3	”	TRANS MEM	59.5	3	519	5′ -GGTGAGGGTGCATCAAATG – 3′	5′ -TTATTCCTTCGTGGCTCTGG – 3′
BDTG 4	”	”	59.8	3	531	5′ -TTGGATCATGTCTCCAACCA – 3′	5′ -ACTTCAGCGCTTGCATGAT – 3′
BDTG 5	”	”	59.8	3	877	5′ -CATCTATCCAACCCCTTACAGC – 3′	5′-CAAGCTTGTTCATGGATTTCAA – 3′
BDTG 6	”	”	60.2	3	1778	5′ -TAGAACTCAGGAGGAGGCATGT – 3′	5′ -TGATTGCGGAAGGATACACA – 3′
BDTG 7	”	”	60.2	3	570	5′ -AGATGGAATGTGTATCCTTCCG – 3′	5′ -GGAAGGATACACCTTCCATTTTC – 3′
BDTG 8	”	LRR	59.5	4	719	5′ -GATGGCTCCTACCGCTATCA – 3′	5′ -GATGTGCAAGAATGGAGCTG – 3′
BDTG 9	”	”	60.9	4	569	5′ -CTCAAATTTAGTGTCTCTGCAGCTC – 3′	5′ -TCCGCGATAGTTAAGCTCTAGG – 3′
BDTG 10	”	”	60	4	735	5′ -TCTGCAAGCACCTCACCTC –3′	5′ -ATGCATTGGAGCGGATTG – 3′
BDTG 11	*xa5*	TF II A	59.9	1	258	5′ -TTCGAGCTCTACCGGAGGT – 3′	5′ -AGAAACCTTGCTCTTGACTTGG – 3′
BDTG 12	”	”	60.2	2	141	5′ -TGTTCTTTTCTCAGGGCCAC – 3′	5′ -AGTTTGGAATCACAGGCCAC – 3′
BDTG 13	*Xa26*	RECP KINASE	59.5	1	594	5′ -GATGCATACTCTTGCTGCCA – 3′	5′ -CAAGACTGTGCAACCCCTG – 3′
BDTG 14	”	”	60.1	1	652	5′ -ACCAGCTATACGGTCCAATCC – 3′	5′ -GCAAGATGCAACCATGAAAGT – 3′
BDTG 15	”	”	59.6	1	616	5′ -CTATTCCTGCTTCTCTTGGCA – 3′	5′ -AGCCTGACGATTTTATCAAGATG – 3′
BDTG 16	”	”	59.6	1	636	5′ -CATCTTGATAAAATCGTCAGGCT – 3′	5′ -GGTTGCACGAAGAAGCTCAT – 3′
BDTG 17	”	”	59.8	1	524	5′ -CGATGATAGCATGTTGGGC – 3′	5′ -AAAAACTATTAAGTACCTGGTGCCAT– 3′
BDTG 18	”	”	59.9	1	567	5′ -TGAGCAGAGTATGGGACTCTAGG – 3′	5′ -ACACCAACTATAAATTGTTGCAGAAC – 3′
BDTG 19	*Xa27*	”	59.9	1	391	5′ -GAAGCCACACACACTGAGACA – 3′	5′ -CGGAGGAGAACTAGAGAGACCA –3′
BDTG 20	*Xa21*	SIGNAL	59.7	1	200	5′ -CACTCCCATTATTGCTCTTCG – 3′	5′ -ACACAACACCCACCCATGT – 3′
BDTG 21	”	LRR	61.8	2	500	5′ -GCTCCTCCAACCTGTCCG – 3′	5′ -TAAACGCTCTTAGAGACGAAAGGT – 3′
BDTG 22	”	”	59.7	2	591	5′ -CAATTCTATCTGGAACCTTTCGTC – 3′	5′-ACCGCTCAAGTTGTTTTCGT – 3′
BDTG 23	”	”	60	2	601	5′ -GGCATTCTACTCGCCTACGA – 3′	5′ -GCATTGCCTTGGATTGAGAT – 3′
BDTG 24	”	CHARGED	59.8	3	707	5′-TGCCTCGATGTTGTCCATTA – 3′	5′ -TCAATGAGGTCCCATCAACA – 3′
BDTG 25	”	KINASE	60.1	4 & 5	1268	5′ -AGGGACAATTGGCTATGCAG – 3′	5′ -AGAATTCAAGGCTCCCACCT – 3′
BDTG 26	*Xa21(A1)*	LRR	59.8	1	280	5′ -TGTTGTTCTCTGCGCTGC – 3′	5′ -CGTCCTGAGGAAGGATAGGTT –3′
BDTG 27	”	”	59.6	1	408	5′ -CATCGCTGGGCAACCTAT – 3′	5′ -TTGGACACGACTTCAAATATGG – 3′
BDTG 28	”	”	59.6	1	397	5′-CCCAGATCCTATTTGGAACATC – 3′	5′ -TGGAAACAGAATCAGGGAGG – 3′
BDTG 29	”	”	59.9	1	410	5′ -AGGTTGCAAATTTGGTGGAG – 3′	5′ -GGAATGCTAAATATTTCAATGGGA – 3′
BDTG 30	”	”	60.2	1	391	5′ -TAGGGCAAATTCCCATTGAA – 3′	5′ -AAAACACCATTGGTTGGCA – 3′
BDTG 31	”	”	59.9	1	405	5′ -CTTTCGTTCAACAGCTTCCAC – 3′	5′ -CACCATCTTGACTATCAAATTCTCC – 3′
BDTG 32	”	”	59.9	1	563	5′ -CTTTCGTTCAACAGCTTCCAC – 3′	5′ -CAATGAAAGGAGGTAGACATAAACAGT – 3′
BDTG 33	”	SNAP	60.2	2	215	5′ -ACTGTTTATGTCTACCTCCTTTCATTG – 3′	5′ -AATAGATTTGCTACGGTCGAACA – 3′
BDTG 34	”	KINASE	59.7	3	363	5′ -TTTGTTATGGAATTCTAGTGTTGGAA – 3′	5′ -CCAACATAACATCAGCATGTCTC – 3′

Gene - Resistance genes from which they were designed; Protein - Protein coded by the DNA sequence amplified by the corresponding marker; Ann Temp – Annealing Temperature of the respective primer pair; Exon no. - Exon of the original gene from which primer pair was designed.

### Isolation of genomic DNA and PCR amplification

Genomic DNA isolation was done according to the method of Walbot [34]. PCR amplification of this DNA was performed with the designed markers. DNA amplification was carried out in 25 μl volumes using 200 μl thin-walled PCR tubes (Axygen, USA) in a MJR thermal cycler. Each reaction mixture contained 100 ng of genomic DNA, 1 μM of each of the two primers, 1× PCR buffer, 1.5mM MgCl_2_ solution, 1mM of dNTP mixture, 1 unit of Taq DNA polymerase and the volume was made up to 25 μl with PCR-grade water. The temperature profile used for PCR amplification comprised 97°C for 5 mins, followed by 35 cycles of 1 min at 95°C, 1 min at 59.5-61.8°C and 2 min at 72°C. The final extension was at 72°C for 10 min.

### Polyacrylamide gel electrophoresis

The PCR products were resolved by native polyacrylamide gel electrophoresis (PAGE) following the protocol given by Sambrook et al. [35], in a 6% gel in vertical electrophoresis tank (gel size of 16 cm × 14 cm, Biotech, India) with Tris-Acetate-EDTA buffer at 150V. The gel, after electrophoresis, was stained with ethidium bromide (5μg of EtBr in 200ml of Tris-Borate-EDTA buffer) washed thoroughly with double distilled water and photographed using a Gel Documentation System (Biorad, USA).

### Allele scoring

Under UV light a cluster of two to five discrete bands (stutter) was apparent in the stained gels for most of the markers. The size (in nucleotides) of the most intensely amplified band was determined using the software Quantity One (Biorad, USA), based on the migration of the band relative to molecular weight size markers (100bp DNA ladder SibEnzyme) included in the gel [36]. The band with the lowest molecular weight for each primer pair was assigned allele number 1 and the progressively heavier bands were assigned incrementally. For any individual primers pair, the presence of an allele in each of the accession was recorded as “1” and the absence of an allele was denoted as “0” [36]. Null alleles were assigned when no amplification product was generated [37]. When an allele was found in less than 5% of the germplasms under study, it was designated as rare [38].

### Genetic relationship analysis using RGA profiles

A 1/0 matrix was constructed for each primer pair using the information of presence or absence of alleles and was used to calculate genetic similarities among the accessions according to Jaccard’s coefficient [39] using NTSYS-pc software package (version 2.02e) [40]. Using pairwise similarity matrix of Jaccard’s coefficient [39] a phylogenetic tree was made by unweighted pair-group method of arithmetic average (UPGMA) and neighbor-joining (NJoin) module of the NTSYS-pc. Support for clusters was evaluated by bootstrap analysis using WinBoot software [41] through generating 1,000 samples by re-sampling with replacement of characters within the 1/0 data matrix. The average polymorphism information content (PIC) was calculated for each primer pair in accordance with the method Anderson *et al.,* [42].

### Sequencing and analysis of polymorphic DNA bands

All the alleles were sequenced. They were eluted using QIAquick Gel Extraction Kit following standard protocol. DNA sequences of the eluted products were determined according to Sanger et al. [43]. Sequencing was done using BioRad sequencer at Bose Institute with a BigDye Terminator v3.1 cycle sequencing kit according to the manufacturer’s manual (Applied Biosystems, Darmstadt, Germany).The sequences were submitted to the NCBI and were analyzed using publicly available software Basic Local Alignment Search Tool, [44] or BLAST, of NCBI (http://www.ncbi.nlm.nih.gov/BLAST/) to find homology. Conserved domains were identified in the sequences using the publicly available software of NCBI conserved domains (http://www.ncbi.nlm.nih.gov/BLAST/).

## Results

### Genetic diversity: number of alleles

The analysis of the PCR profiles of the 41 Caricaceae accessions generated using the 34 RGA primer pairs is summarized in Table 3. Fourteen out of 34 RGA primers used produced polymorphic profiles while the rest of the 20 primer pairs failed to generate amplification products. A total of 115 alleles were produced by the 14 RGA primer pairs; the number of alleles ranging from 4 (BDTG 21) to 10 (BDTG11, BDTG12, BDTG14, BDTG19, BDTG25 and BDTG31). The average number of alleles was 8.375 per locus.

**Table 3:** **Minimum and maximum molecular weight, total number of alleles, rare alleles, null alleles and PIC values for the primers which gave amplification product**

**Marker**	**Gene**	**Protein**	**Min MW in bp**	**Max MW in bp**	**Number of alleles**				**Rare alleles**	**Null alleles**	**PIC values**			
					**Total**	**V&J**	**FA**	**IA**			**Total**	**V&J**	**FA**	**IA**
BDTG11	*xa5*	TF II A	138.77	250.14	10	3	4	9	0	0	0.846	0.449	0.775	0.959
BDTG12	”	”	115	1046	10	5	4	7	4	0	0.801	0.877	0.633	0.909
BDTG13	*Xa26*	RECP KINASE	108	182.99	6	2	2	6	1	1	0.611	0.469	0.408	0.882
BDTG14	”	”	141.28	252.08	10	7	4	10	2	2	0.852	0.939	0.816	0.992
BDTG17	”	”	98	269.81	8	3	5	5	1	1	0.764	0.612	0.878	0.919
BDTG19	*Xa27*	”	173.88	288.77	10	3	5	10	0	0	0.829	0.633	0.878	0.977
BDTG21	*Xa21*	LRR	98	107.70	4	2	3	4	0	0	0.661	0.245	0.633	0.805
BDTG22	”	”	170	590	8	4	1	7	0	1	0.669	0.775	0.878	0.977
BDTG23	”	”	104.27	210.39	8	4	4	8	0	1	0.851	0.714	0.939	0.974
BDTG24	”	CHARGED	176	387.20	6	3	2	5	0	1	0.617	0.714	0.959	0.971
BDTG25	”	KINASE	88.940	197.13	10	5	5	4	4	1	0.728	0.245	0.816	0.992
BDTG30	*Xa1(A1)*	”	210.11	391.46	9	4	5	8	0	1	0.815	0.775	0.249	0.894
BDTG31	”	”	110	650	10	3	2	10	0	0	0.796	0.939	0.775	0.528
BDTG34	”	KINASE	325.66	373.31	6	2	4	5	0	2	0.645	0.816	0.959	0.992
**Total**					115	50	50	102	12	11	10.485	9.202	10.59	12.77
**Average**					8.21	3.57	3.57	7.29	0.86	0.79	0.75	0.66	0.76	0.91

MinMW – Minimum molecular weight of the alleles in that locus, Max MW – Maximum molecular weight of the alleles in that locus, V& J – accessions of *Vasconcellea* and *Jacaratia,* FA – foreign *Carica papaya* accessions, IA - Indian *Carica papaya* accessions.

As a group the total number of alleles for 6 *Vasconcellea* and 1 *Jacaratia* accessions was 50 with an average of 3.57 alleles /locus. The smallest number of alleles identified was 2, amplified by BDTG13, BDTG21 and BDTG34. The highest number of alleles in this category was 7, amplified by BDTG14. The total number of alleles from the 7 foreign *Carica papaya* accessions was also 50 with an average of 3.57 alleles/locus. The lowest number of alleles identified in this category was 1 (amplified by BDTG 22) and the highest was 5 (amplified by BDTG17, BDTG19, BDTG25 and BDTG30). The 27 Indian *Carica papaya* accessions produced 102 alleles with an average of 7.29 alleles/locus. The lowest and highest number of alleles identified in this category was 4 (by markers BDTG 21 and BDTG 25) and 13 (by marker BDTG14) respectively.

When grouped according to the category of motif, the average number of alleles produced by the 14 RGA primer pairs amplifying the LRR motif, the kinase motif, the charged domain and the TFIIA domain were 7.8, 8, 6 and 10 alleles/primer pair respectively.

### Details of the amplification products obtained from the RGA primer pairs

BDTG11 and BDTG12, primer pairs designed from the TF IIA domain of the gene *xa5*, amplified 10 alleles each. The primer BDTG11 was developed from exon 1 and BDTG12 was designed from exon 2 of gene *xa5*. Four rare alleles were identified by BDTG12 and no null alleles were found. The primer pairs BDTG13, BDTG14 and BDTG17 designed from the receptor kinase domain of the *Xa26* gene amplified 12 alleles while the rest of the primer pairs, BDTG15, BDTG16 and BDTG18 failed to amplify. The primers pairs BDTG13 and BDTG17 identified 1 rare and 1 null allele each while BDTG14 identified 2 rare and 2 null alleles. BDTG 19, the primer pair designed from the *Xa27* gene produced 10 alleles and rare or null alleles were absent. Except for the signal sequence, the primer pairs developed from the other regions of the gene *Xa21* amplified 36 alleles (Table 3). Those primer pairs were BDTG21, BDTG22, BDTG23, BDTG24 and BDTG25. BDTG21, designed from LRR domain, exon 2, of gene *Xa21* produced 4 alleles. No rare or null alleles were identified. The primer pair BDTG22, designed from LRR domain, exon 2, of gene *Xa21* produced 8 alleles and 1 null allele. BDTG23 designed from LRR domain, exon 2, of gene *Xa21* produced 8 alleles and a null allele. The primer pair BDTG 24 designed from the charged domain, exon 3 of gene *Xa21* produced 6 alleles and 1 null allele. The primer pair BDTG25 designed from kinase domain of gene *Xa21* produced 10 alleles and 4 rare alleles and 1 null allele. The primer pairs BDTG30, BDTG31 and BDTG34 designed from gene *Xa1(A1)*, produced amplification products, the rest i.e. BDTG 26, BDTG 27, BDTG 28, BDTG 29, BDTG32 and BDTG33 did not produce any amplification product. BDTG30 produced 9 alleles and one null allele. The primer pair BDTG31 produced 10 alleles. No null or rare alleles were produced. The kinase domain (exon 3) of *Xa1(A1)* was amplified by the primer pair BDTG34 and it produced 6 alleles and 1 null allele. The primer pairs for the gene *Xa1* did not amplify any product.

### PIC values

The PIC values, which denote allelic diversity and frequency among germplasms, had an average value of 0.763 per primer pair. The range of PIC value was 0.611 for primer pair BDTG13 to 0.852 for the primer pair BDTG14. That means the most diverse region as well as the region with minimum diversity lies within the same gene. Categorically average PIC value for the *Vasconcellea* accessions was 0.661 per primer pair with a range of 0.245 for primer pair BDTG21 and BDTG25 to 0.939 for primer pairs BDTG14 and BDTG31. For the foreign papaya accessions the average PIC value was 0.716 per primer pair and range of PIC value was 0.245 (BDTG30) to 0.939 (BDTG23). The Indian papaya accessions had an average PIC value of 0.92 per primer pair. The range of PIC value for them was 0.528 (BDTG 31) to 0.992 (BDTG14, BDTG25 and BDTG34). From the PIC values it is evident that allelic diversity is the highest among the Indian papaya accessions. An ANOVA test (Additional file 1: Table S1) was done with the PIC values of the different categories of germplasm. It was proved from that test that the PIC values of the three categories of papaya germplasms used in this study were significantly different from each other.

### Rare and Null alleles

A total of 12 rare alleles were identified with an average of 0.86 rare alleles per loci. The highest number of rare alleles (4 rare alleles) was observed in the profile of the primer pairs BDTG12 and BDTG25. The accession of *Jacaratia spinosa* had 3 rare alleles, *Vasconcellea microcarpa* and *V. parviflora* had 2 while *V. pubescens*, *V. quercifolia* and *V. stipulata* each had one rare allele. The *Carica papaya* accessions Solo 109 and CO1 each had 1 rare allele. A total of 11 null alleles were detected. The primer pairs BDTG14 and BDTG34 each produced 2 null alleles while primer pairs BDTG13, BDTG17, BDTG22, BDTG23, BDTG24, BDTG25 and BDTG30 produced 1 null allele each. The accessions Orissa local had 2 while CO1 and Madhu had 1 null allele each. Seven null alleles were identified amongst the other Caricaceae accessions. *Vasconcellea quercifolia* and *Jacaratia Spinosa* had 2 while *V. goudotiana*, *V. microcarpa* and *V. pubescens* had 1 null allele each.

### Clustering of the Caricaceae accessions

**Figure 1:**
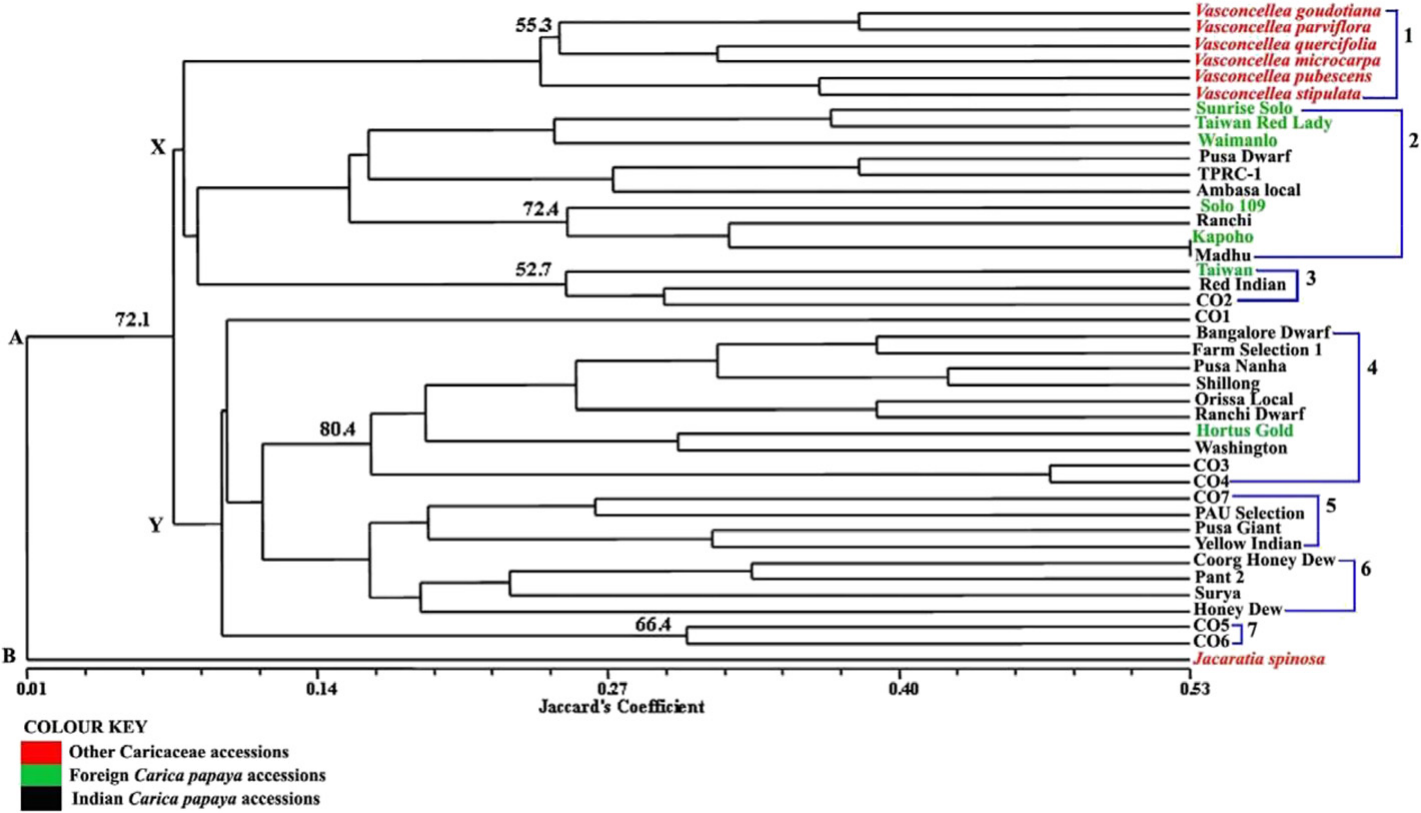
**Dendrogram of 41 Caricaceae genotypes based on Jaccard's genetic similarity coefficient.**

The dendrogram given in Figure 1 was made from genetic similarity values derived from the 1/0 matrix of the RGA profiles (Additional file 2: Table S2 1/0 matrix). The strength of the dendrogram nodes was estimated with a bootstrap analysis using 1000 permutations. The similarity among the Caricaceae accessions ranged from 1% to 53%. Two distinct clusters had separated at 1% level of similarity; “Cluster A”, consisted of 40 accessions and “Cluster B” consisting of just the one accession of *Jacaratia spinosa*. Cluster A was divided into 2 sub-clusters X and Y at 7.5% level of similarity. Both the clusters X and Y underwent further sub-divisions and segregated into 7 smaller clusters at various levels of similarity, as shown in Figure 1. The most significant segregation was at the 24.4% level of similarity at which point all the 6 accessions of *Vasconcellea* separated out from the rest of the accessions. There were two other significant clusters: the cluster separating at 15% similarity consisted of 5 accessions each of the Indian and the foreign caricas, while the cluster separating at 15.9% level of similarity consisted of 9 Indian *Carica papaya* accession and one foreign accession Hortus Gold. The maximum genetic similarity of 53%, was observed between the accessions Kapoho (foreign *Carica papaya*) and Madhu (Indian *Carica papaya*).

### Sequence analysis

The information about the details of homology searches are given in Table 4. A total of 563 sequences were obtained, of which 394 showed significant homology with various sequences of *Oryza sativa.* Out of the 41 DNA sequences amplified by BDTG11 (gene xa5), 35 showed significant homology with *Oryza sativa* Indica Group cultivar IRGC 16339 *xa5* gene, partial cds. Out of the 31 sequences amplified by BDTG12, ten were allotted accession numbers by NCBI. The sequences JM426511.1 (from *Vasconcellea parviflora*), JM426525 (from *Vasconcellea stipulata*), JM426506 (from *Vasconcellea quercifolia*), JM426460 (from CO5), JM426516 (from Bangalore Dwarf) were significantly homologous to *Oryza nivara* cultivar 106133 XA5 (*xa5*) gene, complete cds JM426495 (from Pusa Nanha) and HR614236 (from CO1) were significantly homologous to *Oryza sativa* Japonica Group Os05g0107700 (Os05g0107700) mRNA. The sequences JM170468 (from Pusa Giant), JM170470 (from *Vasconcellea pubescens*) and JM170472 (from Shillong) were significantly homologous to sequence of *Oryza sativa* Japonica Group Os08g0280600 (Os08g0280600) mRNA. The other 21 sequences derived from the PCR profiles of BDTG12 (gene *xa5*) were significantly homologous to the sequence *Oryza sativa* Indica Group cultivar IRGC 27045 *xa5* gene. Among the sequences amplified by the BDTG12, JM426506 (from *Vasconcellea quercifolia)* and JM426460 (from CO5) showed significant homology with conserved domain of Gamma subunit of transcription initiation factor IIA. The sequence JM426495 (from Pusa Nanha) showed significant homology with conserved domain of Cytochrome P450. The sequence JM170472 (from Shillong) showed significant homology with the conserved domain of Methyl-accepting chemotaxis protein (MCP) signaling domain. Out of the sequences amplified by the primer BDTG13, 33 sequences were significantly homologous to the sequence *Oryza sativa* isolate BDTG13-Bhasa receptor kinase (*Xa26*) gene and one, HR614235.1 (from CO1) showed homology with the sequence *Oryza sativa* Japonica Group Os03g0579200 (Os03g0579200) mRNA, complete cds. The sequence HR614235.1 (from CO1) was also significantly homologous to the conserved domain of Nickel-dependent hydrogenase. The sequences amplified by the primer pair BDTG17 were significantly homologous to the sequence of *Oryza sativa* (japonica cultivar-group) bacterial blight resistance protein XA26 *(Xa26 gene)*, complete cds. Some of the sequences were also homologous to the conserved domain of LRR receptor-like protein kinase. The sequences amplified by the primer pair BDTG21 were significantly homologous to the sequence of *Oryza sativa* Indica Group *Xa21* gene for receptor kinase-like protein, complete cds, cultivar: Zheda8220. The conserved domains of LRR could be identified within these sequences. The sequences amplified by the primer pairs BDTG22, BDTG23, BDTG24 and BDTG25 respectively were significantly homologous to the sequences of *Oryza rufipogonXa21F* pseudogene. The sequences amplified by the primer pairs BDTG30 and BDTG31 were significantly homologous to the sequence of *Oryza sativa* Japonica Group Os11g0559200 (Os11g0559200) mRNA. The sequences amplified by the marker BDTG30 were significantly homologous to the conserved domains of LRR receptor-like protein kinase. The sequences amplified by the primer pair BDTG34 were significantly homologous to *Oryza longistaminata* receptor kinase-like protein gene family. The sequences amplified by the primer pairs BDTG14 and BDTG19 did not show any significant homology.

**Table 4:** **Details of homology of the DNA sequences identified in this study**

**Marker**	**N**	**N1**	**GenBank Acc. No.**	**Genotype name of the GenBank accession**	**L**	**BLAST homology searches (Megablast)**		**Q**	**Conserved domain homology searches**	
						**Homology**	**E-value**		**Homology**	**E- value**
BDTG 11	41	31	Not assigned	Not applicable	Average length 215bp	*Oryza sativa* Indica Group cultivar IRGC 16339 *xa5* gene, partial cds	4e-56	80%	Not found	Not applicable
BDTG 12	41	31	Not assigned	Not applicable	Average length 456bp	*Oryza sativa* Indica Group cultivar IRGC 27045 *xa5* gene	2e-135	80%	Not found	Not applicable
		10	JM426511.1	*Vasconcellea parviflora*	189	*Oryza nivara cultivar 106133 XA5 (xa5) gene, complete cds*	1e-23	47%	Not found	Not applicable
			JM426525	*Vasconcellea stipulata*	215		5e-19	35%	Not found	Not applicable
			JM426506	*Vasconcellea quercifolia*	123		4e-29	77%	Gamma subunit of transcription initiation factor IIA	1.54e-04
			JM426460	CO5	123		1e-17	73%	Gamma subunit of transcription initiation factor IIA	6.11e-04
			JM426516	Bangalore Dwarf	115		1e-16	80%	Not found	Not applicable
			JM426495	Pusa Nanha	573	*Carica papaya BAC clone 90D06, complete sequence* mRNA	2e-24	17%	Cytochrome P450	3.08e-24
			HR614236	CO 1	1046	Brassica rapa subsp. pekinensis clone KBrH011C10, complete sequence	7e-64	30%	Serpentine type 7TM GPCR chemoreceptor Srz	1.74e-04
			JM170468	Pusa Giant	281	No significant similarity found	Not found	Not found	Not found	Not applicable
			JM170470	*Vasconcellea pubescens*	275		Not found	Not found	Not found	Not applicable
			JM170472	*Vasconcellea pubescens*	555	Pseudomonas pseudoalcaligenes CECT 5344 complete genome	4e-138	70%	Methyl-accepting chemotaxis protein (MCP), signaling domain	7.37e-33
BDTG 13	40	34	Not assigned	Not applicable	Average length 175	*Oryza sativa* isolate BDTG13-Bhasa receptor kinase (*Xa26*) gene	1e-16	79%	Not found	Not applicable
BDTG 13		1	HR614235.1	CO1	108	*Carica papaya* chloroplast, complete genome	0.080	89%	Nickel-dependent hydrogenase	1.74e-16
BDTG14	39	0	Not assigned	Not applicable	Average length 232bp	No significant similarity found	Not found	Not found	Not found	Not found
BDTG17	40	31	Not assigned	Not applicable	Average length 256bp	Oryza sativa (japonica cultivar-group) bacterial blight resistance protein XA26 *(Xa26)* gene, complete cds	3e-162	55%	LRR receptor-like protein kinase	1.23e-05
BDTG19	41	0	Not assigned	Not applicable	Average length 252bp	No significant similarity found	Not found	Not found	Not found	Not applicable
BDTG21	41	30	Not assigned	Not applicable	Average length 105bp	*Oryza sativa* Indica Group *Xa21* gene for receptor kinase-like protein, complete cds, cultivar:zheda8220	4e-161	76%	LRR	6.45e-07
BDTG22	40	37	Not assigned	Not applicable	Average length 367bp	*Oryza rufipogon* *Xa21F* pseudogene, strain:W1236	0.0	82%	Not found	Not applicable
BDTG23	40	31	Not assigned	Not applicable	Average length 203bp		0.0	80%	Not found	Not applicable
BDTG24	40	29	Not assigned	Not applicable	Average length 287bp	*Oryza rufipogon* *Xa21F* pseudogene, strain:W149	0.0	80%	Not found	Not applicable
BDTG25	40	35	Not assigned	Not applicable	Average length 181bp	*Oryza rufipogon* *Xa21F* pseudogene, strain:W593	0.0	79%	Not found	Not applicable
BDTG30	40	35	Not assigned	Not applicable	Average length 254bp	*Oryza sativa* Japonica Group Os11g0559200 (Os11g0559200) mRNA	2e-137	72%	LRR receptor-like protein kinase	3.69e-11
BDTG31	41	34	Not assigned	Not applicable	Average length 362bp		1e-173	65%	Not found	Not applicable
BDTG34	39	25	Not assigned	Not applicable	Average length 347bp	*Oryza longistaminata* receptor kinase-like protein gene, family	2e-110	70%	Not found	Not applicable

N – Total number of sequences obtained.

N1 – Total number of sequences producing significant homology with various sequences of *Oryza sativa.*

N2 – Total number of sequences allotted accession number by NCBI Genbank.

L – length of the sequence in bp.

Q – percentage of query coverage.

## Discussion

According to Nordborg and Weigel [45] genomic potential and its association with phenotypic variation of any plant species can be achieved by documentation of genomic polymorphism at specific loci controlling various traits using specific genomic region based primers. This variation then has to be coupled with association mapping, a method popularly known as Genome Wide Association mapping. In this study we have used 34 pairs of primers [33] developed from conserved domains of 6 BLB resistance genes of rice, to detect the presence of amplified DNA bands (RGAs) and their polymorphism in a set of 41 Caricaceae accessions. Of these 34 primer pairs, 14 gave amplification profiles in this set of accessions. Since the primers were originally designed to amplify conserved domains of rice BLB resistance genes, they are not expected to behave as random primers and will only amplify sequences with a certain degree of stringency. Apart from clear and consistent amplification profiles, stutter bands, i.e. minor PCR products of lower intensity and lacking or having extra repeat units than the main allele, [46] were also present in the profiles of most of the markers used. Null alleles were present probably due to mutations in the binding region of one or both of the primers, thereby inhibiting primer annealing [37].

In the dendrogram (Figure 1) the accessions of *Vasconcellea* species and *Jacaratia spinosa* had segregated from the *Carica papaya* accessions into different clusters. The *Vasconcellea* accessions had 7.5% similarity with the *Carica papaya* accessions whereas *Jacaratia spinosa* had only 1% similarity with either *Carica papaya* or *Vasconcellea* accessions. As indicated in a previous publication by Sengupta et al., [47] this finding was similar to that proposed by taxonomic descriptions of Badillo [48] and Amplified Fragment Length Polymorphism (AFLP) study of Van Droogenbroeck et al. [49]. Probably due to their similar lineage, the foreign *Carica papaya* accessions Sunrise Solo, Solo 109, Kapoho and Waimanalo had grouped into the same sub cluster (sub cluster 2). Such grouping was also obtained using the SSR profiles in a previous study [50]. The Indian *Carica papaya* accessions Pusa Dwarf, RT1, Ambasa local, Ranchi and Madhu were included in the same cluster as Sunrise Solo in the dendrogram of Figure 1. This indicates a similar genetic nature of the concerned loci amplified by the primers used in this study. Whether those Indian *Carica papaya* accessions share the same lineage with the foreign *Carica papaya* accessions is not known because their parentage has not been elucidated. The accessions of the Coimbatore varieties (CO1-CO7) are phenotypically distinct and were bred at Tamil Nadu Agricultural University by different workers [50]. Like the dendrogram obtained using SSR profiles [47], these accessions have segregated into different sub clusters in this case as well. These trends were also reiterated in a dendrogram derived from the combination of the SSR profiles and the RGA profiles (Additional file 3: Figure S1). In could be observed from that dendrogram (Figure 1) that *Jacaratia spinosa* had segregated out as a separate cluster all by itself and is only 2% similar with the rest of the Caricaceae accessions. In previous taxonomic classifications the genus *Carica* L. was divided into two sections, *Carica* and *Vasconcellea.* This segregation was based on the number of locules in the ovary as well as other morphological similarities between the two sections Based on genetic and morphological characteristics respectively Aradhya et al., [51] and Badillo [48] had separated the two sections into two different genera *Vasconcellea* Saint-Hilaire and *Carica.* According to the findings of Aradhya et al. [51], Olson [52,53] and Kyndt et al [54] there is a possibility that *Jacaratia* shares a common ancestor with, or lies at the origin of *Vasconcellea* but not of *Carica.* In our dendrogram we see that *Vasconcellea*, *Carica* and *Jacaratia* have formed 3 distinct clusters. Moreover the similarity between *Vasconcellea* and *Carica* is more than the similarity between these two genus and *Jacaratia spinosa*. In our previous study of genetic diversity analysis with SSR [47], *Vasconcellea* and *Jacartia* were placed in the same cluster and *Carica* had segregated as a separate cluster. However in this case the alleles of the concerned loci were more similar between *Vasconcellea* and *Carica* hence they have been brought together and *Jacaratia* has separated as an outgroup.

In the same dendrogram of Additional file 3: Figure S1, accessions of *Vasconcellea sp.* along with Hortus Gold formed a separate sub cluster. The foreign *Carica papaya* accessions Solo109, Sunrise Solo, Kapoho and Waimanlo had grouped together in a single sub cluster. The accessions of the Coimbatore varieties (CO1 – CO7) and the Pusa Giant, Pusa Dwarf and Pusa Nanha have segregated into different sub clusters.

The conserved domains identified in the sequences were gamma subunit of transcription initiation factor IIA, Cytochrome P450, MCP, signaling domain, Nickel-dependent hydrogenase, LRR receptor-like protein kinase and LRRs. Out of these the LRR domain is present both in pathogen-associated molecular patterns (PAMP) receptors, and in majority of Resistance (R) proteins [55]. Some R proteins structurally resemble the PAMP receptor like kinases (RLKs), such as the rice *Xa21* and *Xa26* proteins [56]. LRR ribonuclease inhibitor (RI)-like subfamily are 20-29 residue sequence motifs present in many proteins that participate in protein-protein interactions and have different functions and cellular locations. A number of LRRs have been identified in this study, but the detailed structure, function and cellular location are not known and will be elucidated in future dissertations.

The sequences JM426506 and JM426460 amplified by the primer BDTG12 showed significant homology with the conserved domain of gamma subunit of transcription initiation factor IIA (TFIIAγ). The primer pair BDTG 12 was designed from the rice gene *xa5*. The mRNA transcribed by the gene *xa5* translates to a protein which acts both as a transcription factor and a bacterial blight resistance protein in rice [57]. TFIIAγ is one of the general transcription factors for RNA polymerase II which increases the affinity of the TATA-binding protein (TBP) for DNA, in order to assemble the initiation complex. TFIIA also functions as an activator during development and differentiation, and is involved in transcription from TATA-less promoters (NCBI). The *xa5* gene is unusual in that it is recessive and does not conform to one of the typical resistance gene structural classes [57]. Whether the xa5-like sequences identified in Caricaceae confers resistance to bacterial diseases or acts simply as a transcription factor is yet to be elucidated.

The sequence JM426495 amplified by the primer BDTG12 showed significant homology with the conserved domain of cytochrome P450 Among the cytochrome P450 enzymes, CYP51 sterol demethylases are one the most ancient and conserved [58]. Apart from its regular function in plants in the synthesis of essential sterols, CYP51 is used for the production of antimicrobial compounds (avenacins) that confer *Fusarium* rot resistance in oats [59]. *Fusarium* rot has previously been reported in papaya by Guevara et al. [60] and Correia et al. [61] and antifungal activity in leaves and seeds of *Carica papaya* L. cv. Maradol due to the presence of triterpenoid glycoside type saponins have already been proposed by Quintal et al. [62]. Perhaps the cytochrome P450 domain identified in our papaya samples also serves a similar function in the production of plant defense compounds.

The sequence JM170472 amplified by the marker BDTG12 was significantly homologous to Methyl-accepting chemotaxis protein (MCP), signaling domain. The cytokinin inducible genes IBC6 and IBC7, identified by Brandstatter and Kieber [63] from etiolated Arabidopsis. They encode proteins similar to Bacterial Response Regulators. The deduced amino acid sequence of IBC6 and IBC7 aligned significantly with the sequence of conserved regions of chemotaxis response regulators CheY from Escherichia coli. The CheY are commonly known as methyl-accepting chemotaxis proteins (MCPs), [64]. However no significant homology was observed between the sequence JM170472 and the sequences of IBC6 or IBC7 or CheY.

The sequence HR614235.1 amplified by the primer pair BDTG13 was significantly homologous with the conserved domain of Nickel-dependent hydrogenase. These enzymes indirectly influence plant productivity through its role in nitrogen-fixing symbionts [65]. A role for nickel in plant disease resistance has also been observed and has been attributed to a direct phyto-sanitary effect on pathogens, or to a role of nickel on plant disease resistance mechanisms [66,67]. The presence of nickel in the bark of *Carica papaya* have already detected by Mishra *et al.,* [68]. However the mechanism of this nickel in disease resistance is yet to be elucidated.

Information on disease resistance genes of papaya is scarce as compared to *Arabidopsis* and *Oryza.* Studies like this one pave way for the vast amount of work yet undone. According to existing reports [https://www.apsnet.org/publications/commonnames/Pages/Papaya.aspx] *Xanthomonas oryzae* is not pathogenic to *Carica papaya* or *Vasconcellea sp*. However Papaya fruits are frequently spoiled by soft rot caused by *Xanthomonas campestris* [69] under post harvest condition. There are no reports of pathogenicity of *Xanthomonas campestris* in Caricaceae under field conditions. Nevertheless the causal organisms of more destructive bacterial diseases of papaya like canker, leaf spot and internal yellowing, *Erwinia sp., Pseudomonas carica-papayae* and *Enterobacter cloacae* respectively are also gammaproteobacteria like *Xanthomonas*. Since the plant disease resistance genes are structurally and functionally conserved, there are possibilities that defence against the pathogenocity of *Erwinia sp., Pseudomonas carica-papayae* and *Enterobacter cloacae* are also mediated in a way similar to that against *Xanthomonas oryzae* in rice. Whether the identified DNA sequences from this study actually have any association with the soft rot disease or any other bacterial disease of papaya are yet to be unfolded. Such experiments were beyond the scope of this study and will be pursued by us in our future endeavors. Primers designed from known disease resistance genes from other plants should also be used to search for homologous DNA bands and sequences. There should be a large scale investigation on the LRR regions of *Carica papaya* and other Caricaceae genus specially *Vasconcellea*. Their uniqueness has already been shown in-silico by Porter et al., [9] and there are chances that DNA sequence analysis of LRR regions will bring forth some more special features. Cloning, characterization and expression analysis of the linked genes or DNA sequences should follow next.

## Conclusion

Several researchers have proved that plant disease resistance genes are structurally and functionally conserved. Based on that principle this study has used 34 primer pairs designed from the conserved domains of 6 BLB resistance genes of rice to identify RGAs in accessions of Caricaceae. Several DNA bands were amplified by 14 primer pairs. The homology of the sequences of the amplified DNA bands with that of Oryza sativa clearly shows that some of the conserved regions of resistance genes are conserved across evolutionary distances between Caricaceae and Oryza while some others are not. The findings of this study should be informative for the elucidating the structure, function and genetic diversity of disease resistance genes of *Carica papaya* and other related species in future.

### Availability of supporting data

The data set supporting the results of this article is included within the additionl file named Additional file 2: Table S2. 1/0 matrix.

## Additional files

Additional file 1: Table S1. Analysis of Variance Table. Additional file 2: Table S2. One/zero matrix for the RGA profiles. Additional file 3: Figure S1. Dendrogram of 41 Caricaceae accessions using SSR and RGA profiles based on Jaccard’s genetic similarity coefficient.
