# Supplementary material for: Genetic diversity analysis in a set of Caricaceae accessions using resistance gene analogues
Source: BMC Genet. 2014 Dec 10;15:137. doi: 10.1186/s12863-014-0137-0 (PMC4271346; doi:10.1186/s12863-014-0137-0)
Supplement: Additional file 1: Table S1. — Analysis of Variance Table. [file 12863_2014_137_MOESM1_ESM.doc]

| **Source of variation** | **Degrees of freedom** | **Sum of Squares** | **Mean Sum Of Squares** | **F value observed** | **F value tabulated at 5%** | **F value tabulated at 1%** |
| --- | --- | --- | --- | --- | --- | --- |
| Category of germplasm | 2 | -50.0492 | -25.0246 | 2721.702 | 3 | 4.61 |
| Number of primers | 13 | 1.701079 | 0.130852 | -14.2316 | 1 | 1 |
| Error | 26 | -0.23906 | -0.00919 |  |  |  |

**Supplementary Table 1. Analysis of Variance Table**

calculation of critical difference

Square root of Error mean square = 0.0958

t value at 0.025 at error df = 2.06

n = 14

so critical difference is 1.044168268

Mod value of

Total of PIC value of (*Vasconcellea* and *Jacartia* accessions) - Total of PIC value of Foreign *Carica papaya* accessions is 1.394

Total of PIC value of (*Vasconcellea* and *Jacartia* accessions) - Total of PIC value of Indian *Carica papaya* accessions is 3.569

Total of PIC value of Foreign *Carica papaya* accessions - Total of PIC value of Indian *Carica papaya* accessions is 2.175

since all the mod values are greater than the cd, the differences are significant.
