# Supplementary figures and images for: Genetic diversity analysis in a set of Caricaceae accessions using resistance gene analogues
Source: BMC Genet. 2014 Dec 10;15:137. doi: 10.1186/s12863-014-0137-0 (PMC4271346; doi:10.1186/s12863-014-0137-0)

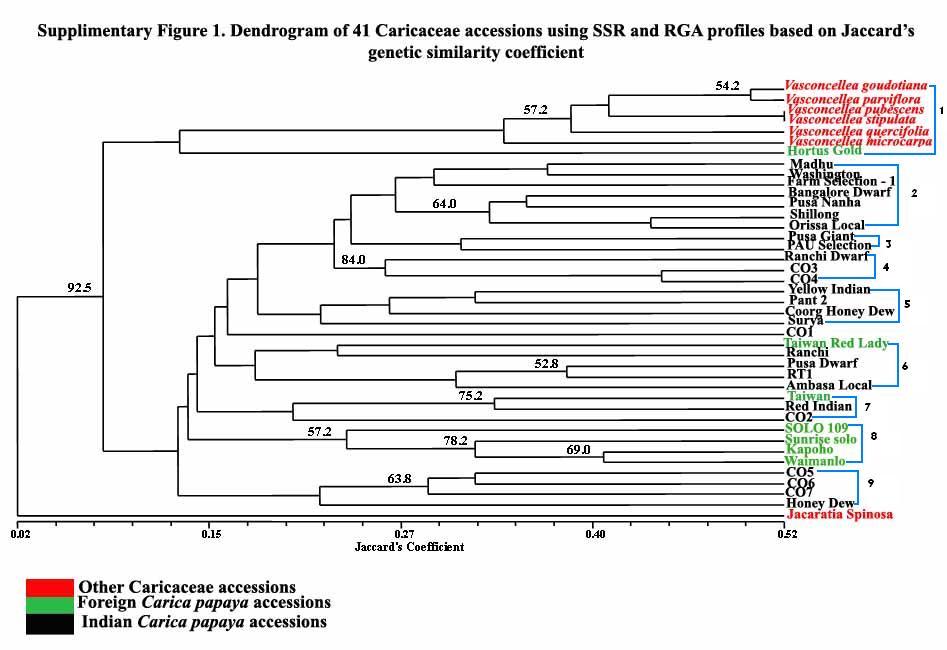

Supplement: Additional file 3: Figure S1. — Dendrogram of 41 Caricaceae accessions using SSR and RGA profiles based on Jaccard’s genetic similarity coefficient. [file 12863_2014_137_MOESM3_ESM.jpeg]
